# Assessment of Total Fat and Fatty Acids in Walnuts Using Near-Infrared Hyperspectral Imaging

**DOI:** 10.3389/fpls.2021.729880

**Published:** 2021-09-09

**Authors:** Julio Nogales-Bueno, Berta Baca-Bocanegra, José Miguel Hernández-Hierro, Raquel Garcia, João Mota Barroso, Francisco José Heredia, Ana Elisa Rato

**Affiliations:** ^1^MED – Mediterranean Institute for Agriculture, Environment and Development & Departamento de Fitotecnia, Escola de Ciências e Tecnologia, Universidade de Évora, Pólo da Mitra, Évora, Portugal; ^2^Food Colour and Quality Laboratory, Facultad de Farmacia, Universidad de Sevilla, Sevilla, Spain; ^3^Departamento de Química Analítica, Facultad de Farmacia, Universidad de Sevilla, Sevilla, Spain

**Keywords:** walnut, total fat, fatty acids, hyperspectral imaging, near infrared, chemometrics

## Abstract

“Persian” walnut (*Juglans Regia* L.) is one of the most consumed tree nuts in the world. It is rich in several bioactive compounds, with polyunsaturated and monounsaturated fatty acids (PUFA and MUFA) appearing at high concentrations. Walnut consumption protects against cardiovascular, carcinogenic, and neurological disorders. The fatty acid profile has usually been determined by gas chromatography, a reliable and robust tool, but also complex, polluting, and time consuming. In this study, near infrared hyperspectral imaging has been used for the screening of total fat, MUFA, PUFA, saturated, and individual fatty acids in walnuts. Five different walnuts varieties have been considered and modified partial least square (MPLS) regressions have been performed. The SEs of prediction (SEP) in external validation (ranged from 2.12% for PUFA to 13.08% for MUFA) suggest that hyperspectral imaging can be a reliable tool for controlling these parameters in a simple, non-destructive and environmentally friendly way.

## Introduction

“Persian” walnut (*Juglans Regia* L.) has been consumed since pre-agricultural times, and cultivated since the ancient Greek empire. Archaeological evidence suggest that Greeks already were aware of the nutritional properties of walnuts (McGranahan and Leslie, 2012; Hayes et al., 2016). Currently, walnut is one of the most consumed nuts in the world. There were cultivated 870,000 metric tons of walnut kernels in the 2017/18 season, which meant an increment over the prior 10-year average of 44% [International Nut and Dried Fruit Council (INC), 2018].

In addition to the walnut’s great sensory attributes, the increase in popularity of this tree nut is due to a number of evidences of being a healthy product. It has been reported a large amount of studies describing different health profits of walnut fruit and emphasizing the protection against the development of cardiovascular-related diseases, age-related neurological disorders, and some cancer types (Hayes et al., 2016). The inclusion of walnut unsaturated fatty acids in the human diet improves endothelium-dependent vasodilation in hypercholesterolemic subjects (Ros et al., 2004), decreases low density lipoprotein cholesterol and blood pressure, decreases both oxidative stress and some markers of inflammation, and increases cholesterol efflux (Kris-Etherton, 2014). Moreover, walnut ingestion has also been linked to benefits to brain health, reducing the oxidant and inflammatory load on brain cells, improving interneuron signaling, increasing neurogenesis, and enhancing sequestration of insoluble toxic protein aggregates (Poulose et al., 2014). Additionally, the incorporation of walnut into mice diet has shown a positive influence (reducing the grown rate or the number of gland tumors) in the development of different human cancers, such as breast, prostate, colon, and renal cancers (Hardman, 2014; Guan et al., 2018).

These positive health effects are mainly due to the unique composition of walnuts, a whole food rich in several nutrients and phytochemicals. Among them, walnuts are rich in some bioactive compounds, such as hydrolysable tannins (gallotannins and ellagitannins), vitamins A and C, vitamin E and other tocopherols, amino acids, etc. In addition, the main nutrients of the walnut are: proteins, carbohydrates, dietary fiber, minerals and, to a greater extent, lipids (Red BEDCA, 2019; U.S. Department of Agriculture, 2019). One of the most important characteristics of walnuts is their lipid composition. Comparing with most other nuts, walnuts show the lowest ratio of saturated fatty acids (SFA) to total fatty acids. Moreover, walnuts have the lower percentage of monounsaturated fatty acids (MUFA) and the larger percentage of polyunsaturated fatty acids (PUFA). Regarding PUFAs, walnut shows a particularly high ω3:ω6 ratio, mainly due to its concentrations of linoleic and α-linolenic acids (Pereira et al., 2008; Hayes et al., 2016), which greatly enhance the health benefits of the nut fruit (Pejin et al., 2012).

Regardless the matrix, fatty acid profile is typically obtained by means of gas chromatography (Pereira et al., 2008; Seidler-Lozykowska et al., 2010; Milinovic et al., 2019). For this purpose, the sample must be minced, and oil must be extracted, preferably at room temperature. Then, an oil sample is subjected to a trans-esterification with methanolic potassium hydroxide, and the generated fatty acid methyl esters (FAME) are evaluated in the chromatographic system (IOC, 2017). In this way, fatty acid profile can be obtained in a precise way. However, this method requires the destruction of the sample, the use of contaminant reagents and a considerable amount of time and other resources.

Similarly to what occurs with other matrixes and analytes, the development of non-destructive, non-polluting and rapid methods for the walnut quality control is of great interest. For example, spectroscopic methods have been successfully applied to different walnut matrices in order to develop regional or varietal discriminations (Peng et al., 2017; Gu et al., 2018; Nogales-Bueno et al., 2020), to identify quality problems (Jensen et al., 2001; Ercisli et al., 2012; Zhao et al., 2018), or to control some nutrients, such as moisture, protein, and fat (Yi et al., 2017). To our knowledge, the application of spectroscopic methods for the screening of the fatty acid profile in walnut has not been not reported, but for other matrices, namely olive oil and passion fruit oil is already described by some authors (Kiefer et al., 2019; Milinovic et al., 2019), or even in other tree nuts (Teixeira and Sousa, 2019). For that reason, the main aim of the present study is to study the availability of the use of a spectral technique [near infrared (NIR) hyperspectral imaging] on the screening of the lipid fraction in walnut fruit. For that purpose, spectra of walnut kernels from five different varieties were acquired with a hyperspectral device, and a sample selection was carried out in order to identify a reduced number of representative samples. Then, the lipid profile of the selected walnuts was obtained and spectroscopic methods for the prediction of total fat and saturated, monounsaturated, polyunsaturated, and individual fatty acids were developed.

## Materials and Methods

### Samples

Walnut samples were provided by a fruit producer’s association located in Alentejo, Fruteco (Borba, Portugal), in the 2018–2019 season. Samples for this study were obtained from five different varieties: “Chandler”, “Franquette”, “Howard”, “Lara”, and “Tulare”. Forty in-shell walnuts were taken for each variety (i.e., a total of 200 samples). They were carried to the laboratory where the walnut shells were removed. Walnut kernels were subjected to a visual inspection in order to identify samples with defects, such as insect damage, fungal growth, abnormal coloration, or detrimental disorders. Defective samples were discarded, and the remaining samples were stored at 4°C until spectra were acquired.

### Hyperspectral Imaging and NIR Data Extraction

Walnut kernels were tempered and submitted to the acquisition of their NIR spectra. For that purpose, a NIR hyperspectral device was used. This device consists in a camera (Xenics Infrared Solutions, Inc., Leuven, Belgium), a spectrograph (Spectral Imaging Ltd., Oulu, Finland), two 70W tungsten iodine halogen lamps (Prilux®, Barcelona, Spain), a mirror scanner (Spectral Imaging Ltd., Oulu, Finland), and a computer system. Hyperspectral images covered the spectral range between 900 and 1,700nm with a spectral resolution of 3.25nm. The procedure performed for NIR spectra acquisition is based on Nogales-Bueno et al. (2020), with some modifications. Briefly, groups of 10 walnut kernels were disposed over a polyethylene tray and under the hyperspectral camera each time. Images were recorded using a time of exposition of 9ms and a frame rate of 50Hz. Two images were acquired for each 10-walnut group. Between both images, walnuts were rotated 180° aiming to acquire as much spectral information as possible. Hyperspectral device was controlled using the instrument acquisition software SpectralDAQ v. 3.62 (Spectral Imaging Ltd., Oulu, Finland).

Reflectance spectra were corrected following a two-point calibration. To this end, in each image acquisition session, two extra images were acquired: (1) a dark current spectrum, where lamps were turned off and the entry window of the mirror scanner was covered and (2) a white reference spectrum, where it was acquired the spectrum of a polytetrafluoroethylene tile (Labsphere Inc., North Sutton, United States) at operation conditions. Then, regions of interest (ROIs) were extracted from the images by means of a segmentation procedure. The segmentation algorithm can identify walnut kernels and background pixels and save only the reflectance spectra of the walnut. For the development of this algorithm, a number of walnut and background spectra were manually extracted from the calibrated images. A stepwise linear discriminant analysis (LDA) was carried out using NIR wavelengths as dependent variables and the membership of the spectra to kernel or background as categorical variable. Then, the LDA model was applied to all the calibrated images and individual walnut spectra were extracted. Noisy wavelengths were found at both extremes of the spectral range and only the subrange 950–1,650nm was kept. Finally, the spectral matrix was transformed to absorbance values and saved. Calibration and segmentation processes were carried out in the software Matlab (R2018a; TheMathWorks, Inc., MA, United States), ENVI 4.7 (ITT Corporation, White Plains, NY, United States), and SPSS 25.0 (SPSS, Inc., Chicago, IL, United States).

**Figure 1:**
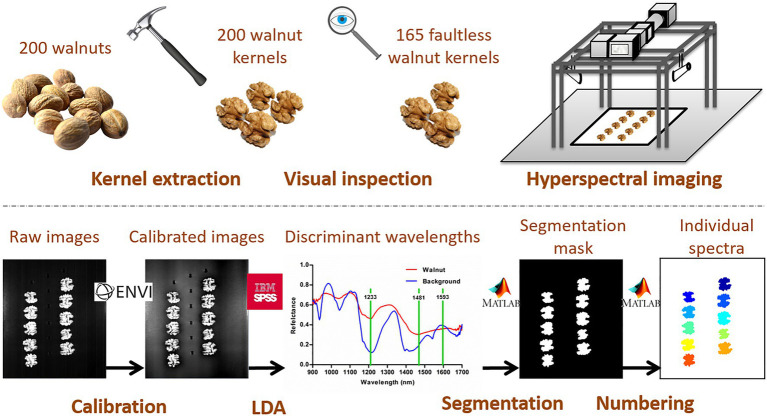
Schematic representation of the entire process between collecting samples and obtaining the spectral matrix: Extraction of walnut kernels, detection of faulty walnuts by visual inspection, hyperspectral imaging acquisition, calibration of raw images, application of LDA for identifying the discrimination wavelengths, segmentation of images, identification and extraction of the individual walnut spectra.

The entire procedure described above has been schematized in Figure 1.

### Sample Selection

Once the walnut kernel spectra were obtained, it was necessary to obtain the chemical values for the reference parameters in these walnuts. However, in order to reduce the number of samples required for the development of spectroscopic methods, a selection of samples was made following the method described by Nogales-Bueno et al. (2015) For that purpose, the spectral information of the samples was analyzed.

A principal component analysis (PCA) was applied to the spectral matrix. Previously, spectra were pre-treated with a standard normal variate (SNV; 2,5,5,1) spectral pre-treatment. Numbers in brackets, respectively represent the number of the derivative, the gap over which the derivative is calculated, the number of data points in a first running average or smoothing, and in a second smoothing (Shenk and Westerhaus, 1995). PCA is an unsupervised pattern recognition tool, which allows studying the latent structure of the matrix. The number of principal components (PC) was established, Mahalanobis distance (H) was measured, and samples were sorted considering their distance to the spectral center. Samples with *H*>3 were identified as spectral outliers and were removed from the spectral matrix. Furthermore, samples were grouped according to their neighborhood Mahalanobis distance (NH). Groups with NH<0.9 were created. Since each group can be considered spectrally homogenous, a calibration sample set was created by selecting one sample for each group. Moreover, by selecting another sample for each group (if possible), a validation set was created. These two sample sets (calibration and validation) were subsequently employed for the development of the spectroscopic methods and for their internal and external validation.

### Reference Parameters (Fatty Acids and Total Fat)

After sample selection, samples allocated into calibration and validation sets were weighted and minced in a mill (IKA® A11 basic) and then they were subjected to the determination of the reference parameters: fatty acid profile and total fat.

#### Fatty Acid Profile

Walnut grounds were moved to 50ml Falcon™ tubes and centrifuged at 26,700 r.c.f (15,000rpm) in a Thermo scientific – Sorvall Lynx 4000 centrifuge for 20min. Centrifugal force helped to extract some oil from walnut grounds at room temperature avoiding fatty acids degradation. For the determination of the fatty acid profile, a modification of the official method for determining these compounds in olive oil (IOC, 2017) was carried out. This method uses a cold methanol solution for producing a trans-esterification of the fatty acids. Briefly, approximately 50mg of the extracted oil were transferred to a glass vial. Then, it was added 2ml of heptane and 0.2ml of methanolic KOH 2M solution. Vials were agitated during 30s in a vortex and, after 5min, when supernatant was clear, this solution was filtered through 0.45μm pore size filters and transferred to chromatographic vials. These vials contained FAME, which were analyzed by gas chromatography equipped with a flame ionization detector (GC-FID).

Analyses were performed in a Hewlett Packard (6890 series) GC-FID equipment using a fused silica capillary column Supelco, SP™ 2380 (60m×0.25mm×0.20μm; Bellefonte PA, United States). The chromatographic method is described in detail in Milinovic et al. (2019). In brief, hydrogen was used as carrier gas with a 1.2mlmin^−1^ flow during the whole runtime (40min). Volume of injection was set at 1μl and Advanced Chromatography Data Station – Clarity Software Solutions v. 7.4, was used for the data acquisition, processing, and instrument control. Fatty acids methyl esters were identified by comparing their retention times with a 37-Component FAME mix standard (Supelco, 10mgml^−1^ of FAME reference standard mixture in methylene chloride) analyzed used the same chromatographic conditions. In addition to individual fatty acids, SFA, MUFA, and PUFA were also evaluated as the sum of the respective peaks.

#### Total Fat

After the centrifugation process, walnut grounds were moved to a glass extraction beaker. All the grounds and fat were collected using petroleum ether as extraction solvent. A total of 150ml of petroleum ether was added to each sample. An automatic Soxhlet apparatus (Soxtherm® SE-416 MK, Gerhardt) was used for performing the total fat extraction. After extraction, solvent was removed and recycled. Total fat was calculated as the sum of the fat used for the fatty acid determination and the fat extracted with the Soxtherm apparatus.

### Data Analysis

In addition of the LDA and PCA, described in the previous sections, other statistical tools were applied to the chemical and spectral data sets.

Modified partial least square (MPLS) regressions were applied for developing calibration methods for the screening of total fat and fatty acid profile. For this purpose, different spectral pre-treatments were applied to the spectra allocated in the calibration set. SNV, multiplicative scattering correction (MSC), detrend, first and second derivatives, and none pre-treatments were tested and only the model with the best results was saved for each reference parameter. MPLS divides the calibration set into a number of subsets in order to perform cross-validation to establish the number of PLS factors and to reduce the possibility of overfitting (Shenk and Westerhaus, 1995). Chemical outliers were identified and removed by the evaluation of the T-statistic and setting the threshold in 2.5units. To do so, residual error is obtained by comparison between the predicted values and the chemical values. Finally, the SE of cross-validation (SECV) is evaluated and expressed as percentage.

Then, the goodness of each MPLS model was tested. To this end, models were applied to the samples allocated into the validation set, and the results were compared to the chemical values previously determined. In this way, a SE of prediction (SEP) in external validation was obtained for each reference parameter. Finally, models were also applied to those samples not selected for calibration or validation sets. Thus, a predicted fatty acid profile was obtained for these samples. Then, these predicted profiles were averaged for each walnut variety and compared to those obtained from the gas chromatography analysis, for samples allocated into the calibration and validation sets. MPLS models were developed and tested with Win ISI® (v1.50; Infrasoft International, LLC, Port. Matilda, PA, United States).

The comparison between the average values for the reference parameters of the different walnut varieties was carried out by means of ANOVA and *post hoc* Tukey test. Total fat, individual fatty acids, SFA, MUFA, and PUFA were used as dependent variables, and walnut variety was used as independent variable or factor. The statistically significant level was considered at *α*=0.05. ANOVA were performed using Statistica v.8.0 software (StatSoft Inc., OK, United States, 2007).

## Results and Discussion

### Sample Sets

Walnut kernels were visually inspected and 35 of the initial 200 samples were identified as defective samples. They were discarded and not taken into account for the rest of this study. The remaining 165 walnut kernels (31 “Chandler”, 29 “Franquette”, 32 “Howard”, 35 “Lara”, and 38 “Tulare” samples) were subjected to the hyperspectral analysis. The segmentation procedure identified three wavelengths (1,233, 1,481 and 1,593nm) for the detection of the ROIs (Figure 1).

Next, the sample selection procedure was carried out. PCA did not identify any spectral outlier, and H and NH distances were measured in a space composed by 12 PCs. These 12 PCs explained the 99% of the spectral variability. Forty-three spectrally equivalent groups were found in this space and one sample for each group was selected and allocated into the calibration set. Therefore, this sample set contained most of the spectral information from the original sample set. Then, another sample of each group was selected, if possible, and allocated into the validation set. In consequence, the 165 initial samples were distributed as follows: calibration set (43 samples), validation set (20 samples), and non-selected set (102 samples).

### Chemical Analysis

Only walnut kernels allocated into the calibration and validation sets were subjected to chemical analysis. These sets of samples comprise 12 samples of “Chandler”, 8 samples of “Franquette”, 4 samples of “Howard”, 23 samples of “Lara”, and 16 samples of “Tulare” varieties. Differences in the number of samples for each variety indicate which varieties are more spectrally homogeneous (“Howard” or “Franquette”) and which are more spectrally heterogeneous (“Lara” or “Tulare”).

Table 1 shows the mean and SEs of the total fat and fatty acids (percent) in each walnut variety. Up to 16 individual fatty acids could be identified and quantified with the chromatographic analysis. Among them, four were SFA (palmitic acid or C16:0, margaric acid or C17:0, stearic acid or C18:0, and heneicosanoic acid or C21:0); eight were MUFA (palmitoleic acid or C16:1 ω7c, hypogeic acid or C16:1 ω9c, cis-10-heptadecenoic acid or C17:1 ω7c, civetic acid or C17:1 ω9c, elaidic acid or C18:1 ω9t, oleic acid or C18:1 ω9c, and vaccenic acid or C18:1 ω7c and gondoic acid or C20:1 ω9); and four were PUFA (linolelaidic acid or C18:2 ω6ct, linoleic acid or C18:2 ω6c, γ-linolenic acid or C18:3 ω6, and α-linolenic acid or C18:3 ω3).

**Table 1:** Mean and SEs of the total fat and fatty acids (percent) in walnut determined by the chemical analysis.

Parameters		Variety				
		Chandler	Franquette	Howard	Lara	Tulare
Total fat		65.58±0.91^a^	68.86±0.68^a^	68.78±2.24^a^	66.91±0.69^a^	66.29±0.95^a^
**Total SFA** ^α^		8.87±0.13^a^	10.17±0.32^b^	8.73±0.37^ac^	9.78±0.13^bc^	9.44±0.24^abc^
**Total MUFA** ^β^		14.44±0.38^a^	16.79±0.51^b^	13.78±1.00^a^	13.62±0.34^a^	13.54±0.48^a^
**Total PUFA** ^χ^		76.69±0.42^a^	73.03±0.56^b^	77.48±1.36^a^	76.60±0.39^a^	77.02±0.63^a^
SFA^α^	**C16:0**	5.96±0.10^a^	7.17±0.27^b^	5.88±0.30^ac^	6.76±0.12^cb^	6.65±0.18^cb^
	C17:0	0.05±0.00^a^	0.06±0.02^a^	0.05±0.00^a^	0.06±0.00^a^	0.06±0.00^a^
	C18:0	2.28±0.08^a^	2.40±0.09^a^	2.25±0.20^a^	2.38±0.07^a^	2.47±0.11^a^
	C21:0	0.57±0.11^a^	0.54±0.30^a^	0.56±0.28^a^	0.58±0.15^a^	0.26±0.02^a^
MUFA^β^	C16:1 ω7c	0.05±0.00^a^	0.07±0.02^a^	0.05±0.00^a^	0.06±0.01^a^	0.06±0.00^a^
	C16:1 ω9c	0.06±0.00^a^	0.16±0.05^a^	0.06±0.01^a^	0.09±0.02^a^	0.07±0.00^a^
	C17:1 ω7c	0.02±0.00^a^	0.02±0.01^a^	0.04±0.02^a^	0.03±0.01^a^	0.02±0.00^a^
	C17:1 ω9c	0.03±0.00^a^	0.03±0.00^a^	0.03±0.00^a^	0.03±0.00^a^	0.03±0.00^a^
	C18:1 ω9t	0.06±0.00^a^	0.06±0.01^a^	0.06±0.01^a^	0.05±0.00^a^	0.05±0.00^a^
	**C18:1 ω9c**	12.98±0.41^ab^	14.84±0.63^b^	12.43±0.90^ab^	12.16±0.37^a^	12.27±0.48^a^
	**C18:1 ω7c**	1.08±0.09^ab^	1.47±0.27^a^	0.99±0.09^ab^	1.01±0.09^ab^	0.93±0.02^b^
	C20:1 ω9	0.14±0.02^a^	0.14±0.05^a^	0.12±0.03^a^	0.17±0.03^a^	0.10±0.01^a^
PUFA^χ^	C18:2 ω6ct	0.07±0.01^a^	0.09±0.03^a^	0.06±0.00^a^	0.07±0.01^a^	0.06±0.00^a^
	**C18:2 ω6c**	62.22±0.51^a^	58.64±0.78^b^	60.36±0.74^ab^	63.74±0.58^a^	62.27±0.76^a^
	C18:3 ω6	0.17±0.04^a^	0.18±0.12^a^	0.14±0.07^a^	0.18±0.05^a^	0.09±0.02^a^
	**C18:3 ω3**	14.23±0.45^ab^	14.12±0.64^ab^	16.92±1.25^b^	12.61±0.45^a^	14.60±0.70^ab^

For each variable, different letters in the same row indicate statistical differences (Tukey test, *α*=0.05). For an easier reading, parameters where significant differences were found have been marked in bold.

α SFA, saturated fatty acids.

β MUFA, monounsaturated fatty acids.

χ PUFA, polyunsaturated fatty acids.

### MPLS Models

After chemically obtaining the total fat and fatty acid profile for the calibration and validation sets, these reference data were assigned, respectively to the correspondent spectral samples. Then, MPLS models for predicting these parameters were developed. MPLS regressions were carried out employing the spectral data of the calibration samples as independent (X) variables and the values of total fat, SFA, MUFA, PUFA, and the individual fatty acids as dependent (Y) variables. Table 2 describes the main descriptors for the MPLS models developed for total fat, SFA, MUFA, and PUFA: the best spectral pre-treatment, the number of samples (N) used to obtain the calibrations after deleting the chemical outliers (T), the number of PLS factors, the range of applicability of the models, and a number of statistical descriptors, which allow describing the goodness of these models. All models present an adequate coefficient of determination (RSQ) and SECV. This error is more noticeable if it is expressed as a percentage of the mean value of the range. In all cases, obtained errors were less than 10%. Afterward, using the spectra and chemical values allocated in the validation set, the developed models were tested and the SEP errors in external validation were obtained (Table 2). The good results obtained for fatty acid families suggested that good MPLS models might be obtained for individual fatty acids. Then, MPLS models were developed for the individual fatty acids identified in the gas chromatography system. Table 3 summarizes the results obtained.

**Table 2:** Main statistical descriptors for the modified partial least square (MPLS) models developed for walnut kernels in the near infrared (NIR) zone close to 950–1,650nm for total fat and saturated monounsaturated and polyunsaturated fatty acids.

Reference parameters	Spectral pretreatments	T outliers	PLS factors	*N* ^a^	Est. Min	*SD* ^b^	Est. Max	SEC^c^	RSQ^d^	SECV^e^	SEP^f^	SECV (%)^e^	SEP (%)^f^
Total fat^g^	MSC 1,5,5,1	3	6	40	56.894	3.417	77.393	0.948	0.923	1.525	2.019	2.272	3.007
SFA^h^	SNV+detrend 2,5,5,1	0	6	43	6.640	0.924	12.186	0.410	0.803	0.750	0.689	7.966	7.320
MUFA^i^	SNV 2,5,5,1	1	7	42	8.644	1.856	19.779	0.835	0.798	1.415	1.860	9.958	13.088
PUFA^j^	MSC 2,10,10,1	2	7	41	69.180	2.404	83.607	1.119	0.784	1.548	1.626	2.026	2.128

a N, number of samples (calibration set).

b SD, standard deviation.

c SEC, standard error of calibration.

d RSQ, coefficient of determination (calibration set).

e SECV, SE of cross-validation (also expressed in percentages with respect to the maximum and minimum estimations).

f SEP, SE of prediction in the external validation (also expressed in percentages with respect to the maximum and minimum estimations).

g Total fat, percentage of total fat extracted from the walnuts.

h SFA, saturated fatty acids (relative percent).

i MUFA, monounsaturated fatty acids (relative percent).

j PUFA, polyunsaturated fatty acids (relative percent).

**Table 3:** Main statistical descriptors for the MPLS models developed for walnut kernels in the NIR zone (950–1,650nm) for the saturated, monounsaturated, and polyunsaturated fatty acids obtained.

Type	Reference parameters	Spectral pretreatments	T outliers	PLS factors	*N* ^a^	Est. Min	*SD* ^b^	Est. Max	SEC^c^	RSQ^d^	SECV^e^	SEP^f^	SECV (%)^e^	SEP (%)^f^
SFA^g^	**C16:0**	SNV+detrend 2,5,5,1	1	6	42	4.588	0.630	8.368	0.283	0.798	0.489	0.663	7.549	10.235
	**C17:0**	SNV 0,0,1,1	4	5	39	0.035	0.005	0.066	0.003	0.668	0.004	0.005	7.730	9.911
	C18:0	MSC 0,0,1,1	1	1	42	1.309	0.352	3.422	0.320	0.173	0.372	0.448	15.705	18.939
	C21:0	SNV 2,10,10,1	4	3	39	0.000	0.192	0.912	0.156	0.342	0.195	0.190	42.644	41.658
MUFA^h^	C16:1 ω7c	MSC 2,5,5,1	3	2	40	0.034	0.006	0.072	0.005	0.330	0.006	0.008	11.731	15.137
	**C16:1 ω9c**	Detrend 2,10,10,1	2	7	41	0.012	0.019	0.127	0.012	0.645	0.016	0.020	23.005	28.756
	C17:1 ω7c	None 0,0,1,1	4	2	39	0.004	0.004	0.031	0.004	0.204	0.004	0.004	23.699	23.121
	C17:1 ω9c	SNV 2,10,10,1	2	2	41	0.021	0.004	0.044	0.003	0.343	0.004	0.005	10.802	15.432
	C18:1 ω9t	SNV 1,5,5,1	5	1	38	0.035	0.006	0.072	0.005	0.292	0.006	0.006	10.242	11.173
	**C18:1 ω9c**	SNV+detrend 2,5,5,1	1	7	42	7.505	1.752	18.019	0.708	0.837	1.395	1.911	10.929	14.974
	C18:1 ω7c	MSC 1,5,5,1	3	3	40	0.618	0.115	1.307	0.083	0.483	0.106	0.162	10.982	16.831
	C20:1 ω9	SNV 1,5,5,1	3	4	40	0.000	0.048	0.257	0.036	0.430	0.045	0.033	35.280	25.701
PUFA^i^	**C18:2 ω6ct**	SNV+detrend 2,5,5,1	2	6	41	0.035	0.009	0.091	0.005	0.699	0.011	0.010	17.025	15.911
	**C18:2 ω6c**	MSC 1,5,5,1	0	7	43	53.100	2.981	70.984	1.816	0.629	3.302	2.981	5.323	4.805
	C18:3 ω6	SNV 2,10,10,1	2	3	41	0.000	0.093	0.396	0.075	0.353	0.088	0.086	44.658	43.445
	**C18:3 ω3**	SNV+detrend 2,5,5,1	2	6	41	7.006	2.313	20.884	1.077	0.783	1.775	1.790	12.758	12.836

Parameters with acceptable RSQ and SEP values have been marked in bold.

a N, number of samples (calibration set).

b SD, standard deviation.

c SEC, SE of calibration.

d RSQ, coefficient of determination (calibration set).

e SECV, SE of cross-validation (also expressed in percentages with respect to the maximum and minimum estimations).

f SEP, SE of prediction in the external validation (also expressed in percentages with respect to the maximum and minimum estimations).

g SFA, saturated fatty acids. They are, respectively: palmitic acid, margaric acid, stearic acid, and heneicosanoic acid.

h MUFA, monounsaturated fatty acid. They are, respectively: palmitoleic acid, hypogeic acid, cis-10-heptadecenoic acid, civetic acid, elaidic acid, oleic acid, vaccenic acid, and gondoic acid.

i PUFA, polyunsaturated fatty acids. They are, respectively: linolelaidic acid, linoleic acid, γ-linolenic acid, and α-linolenic acid.

### Application of the Models

Finally, the values of total fat, SFA, MUFA, PUFA, and individual fatty acids were predicted for the samples not used in the development or validation of the models, i.e., for the samples allocated into the non-selected set (Table 4). Regarding individual fatty acids, only those whose MPLS models had adequate RSQ, SECV, and SEP values were predicted.

**Table 4:** Mean and SEs of the total fat and fatty acids (percent) in walnut obtained by the application of MPLS models to non-selected spectra.

Parameters		Cultivar				
		Chandler	Franquette	Howard	Lara	Tulare
Total fat		66.76±0.38^a^	67.64±0.75^ab^	68.13±0.39^ab^	68.24±0.80^ab^	69.16±0.61^b^
Total SFA^α^		9.06±0.09^a^	9.89±0.12^b^	9.02±0.09^a^	9.89±0.16^b^	9.76±0.15^b^
Total MUFA^β^		14.18±0.23^a^	15.76±0.27^b^	13.91±0.18^a^	13.24±0.40^a^	14.04±0.27^a^
Total PUFA^χ^		76.28±0.24^a^	74.51±0.33^b^	76.97±0.24^a^	76.82±0.62^a^	76.52±0.29^a^
SFA^α^	C16:0	6.12±0.07^a^	6.68±0.06^b^	6.02±0.06^a^	6.77±0.12^b^	6.65±0.09^b^
	C17:0	0.05±0.00^a^	0.04±0.00^b^	0.05±0.00^a^	0.05±0.00^a^	0.05±0.00^a^
MUFA^β^	C16:1 ω9c	0.07±0.00^a^	0.09±0.00^b^	0.07±0.17^a^	0.07±0.01^a^	0.08±0.01^ab^
	C18:1 ω9c	12.59±0.23^a^	13.63±0.27^b^	12.43±0.90^a^	12.08±0.40^a^	12.10±0.29^a^
PUFA^χ^	C18:2 ω6ct	0.06±0.00^ab^	0.07±0.00^b^	0.06±0.00^a^	0.06±0.00^ab^	0.06±0.00^ab^
	C18:2 ω6c	62.50±0.32^ab^	59.83±0.37^c^	59.41±0.32^c^	63.53±0.77^a^	60.88±0.47^bc^
	C18:3 ω3	13.04±0.24^a^	13.88±0.28^a^	15.13±0.32^b^	13.34±0.53^a^	13.32±0.31^a^

Only MPLS models with adequate RSQ and SECV were applied. For each variable, different letters in the same row indicate statistical differences (Tukey test, *α*=0.05).

α SFA, saturated fatty acids.

β MUFA, monounsaturated fatty acids.

χ PUFA, polyunsaturated fatty acids.

## Discussion

### Total Fat and Fatty Acid Profile in Walnuts

Total fat ranged from 65.85% for “Chandler” to 68.86% for “Franquette” varieties. However, there were not found significant differences among varieties. According to the bibliography, walnuts contain an average value of 65% fat by weight (Hayes et al., 2016; Fatima et al., 2018). However, higher and lower values are also described. Muradoglu et al. (2010) reported average values of 50% for total fat content in walnuts, contrarily to Pereira et al. (2008) who reported values between 69 and 72% for total fat content by weight. These differences can be attributed to the influence of the variety, harvesting year and different environmental conditions, which may influence the chemical composition of the fruits. In fact, the oil content, the fatty acids, vitamin E, and tocopherols have been found by others to vary significantly among different walnut cultivars and environmental condition (Amaral et al., 2005).

In this study, the fatty acids compositions of walnut varieties ranged from 8.73 to 10.17% for SFA; from 13.54 to 14.44% for MUFA, and from 73.03 to 77.48% for PUFA. These results are consistent with the fact that walnut is the consumable tree nut with the lowest percentage of SFA and the highest percentage of PUFA. Regarding to individual fatty acids, the 69 and 25% of the SFA were, respectively palmitic and stearic acids and 90% of the MUFA was oleic acid. Also 81 and 19% of the PUFA correspond to linoleic and α-linolenic acid, respectively. Similar results can be widely found for walnuts in the literature (Greve et al., 1992; Amaral et al., 2003; Li et al., 2007; Pereira et al., 2008; Poggetti et al., 2018; Red BEDCA, 2019; U.S. Department of Agriculture, 2019).

Considering differences between walnut varieties, “Franquette” has significantly higher amounts of SFA and MUFA and a lower concentration of PUFA. Similar trends in the fatty acid profile were reported between “Lara” and “Franquette” by other researchers (Amaral et al., 2003; Pereira et al., 2008), between “Franquette” and “Howard” (Poggetti et al., 2018; Cittadini et al., 2020) and also between “Tulare” and “Franquette” (Cittadini et al., 2020). Moreover, similar profiles for “Chandler” and “Howard” varieties were also reported in Greve et al. (1992) and in Cittadini et al. (2020).

The higher amount of SFA in “Franquette” can be easily linked to a greater oxidative stability of walnut oil (Rabrenovic et al., 2011). Furthermore, the higher concentration of MUFA and lower concentration of PUFA leads to a higher MUFA/PUFA ratio in this variety than in the others. In fact, MUFA/PUFA ratio has also been linked to oxidative stability in tree nuts (Sena-Moreno et al., 2016). The higher the MUFA/PUFA ratio, the greater the oxidative stability. Indeed, MUFA/PUFA ratio for “Franquette” variety is 0.23 while for the remaining varieties is 0.18–0.19. Therefore, “Franquette” is more stable than the other varieties, and can be considered a good option for walnut oil production. Nevertheless, it is necessary to take into account the stability problems of walnut oil when compared with other tree nuts, mainly due to the much higher concentration of PUFA in walnut (Savage et al., 1999). In addition, this greater oxidative stability of “Franquette” suggests a better aptitude for the sale of walnut kernel, whereas the remaining varieties are more suitable for in-shell sale. In fact, shells protect the kernel from oxidative agents (Hosseini et al., 2014). On the other hand, a higher concentration of PUFA is linked to greater health profits (Ros et al., 2004; Kris-Etherton, 2014), which suggests that “Franquette” walnuts could have a slightly lower health benefits than other varieties.

### Goodness of the MPLS Models

Standard error of prediction error in external validation is the best indicator of the goodness of the models. SEP ranged from 2.12% for PUFA to 13.08% for MUFA. Also for total fat, the result obtained for SEP was 3.01% which is an expected value since fat is the main component of these samples and the reference method for its determination is relatively simple. However, SEP values obtained for the fatty acid families are remarkable since the reference method used, gas chromatography, has a relatively high complexity. These SEP errors indicate that near infrared hyperspectral imaging can be a reliable tool for the control of these parameters in walnut kernels in a simple, non-destructive and environmentally friendly way. However, it should be taken into consideration that to obtain definitive models it would be necessary to employ more sample sets in order to consider seasonal and regional variations.

Regarding the MPLS models developed for the individual fatty acids identified in the gas chromatography system (Table 3), the first interesting outcome is the number of PLS factors selected by the software in the development of the different MPLS models. Attending the PLS factors, two groups of models can be defined: a first group with few PLS factors (1–4) and a second group with numerous PLS factors (5–7). In MPLS regression, each PLS factor is a compromise, trying to explain spectral variation while trying to correlate highly with the Y variable without overfitting (Shenk and Westerhaus, 1991; Westerhaus, 2014). Therefore, a model with few PLS factors might indicate that this compromise was hardly achievable. In this model, PLS factors mainly describe X variability without describing Y. The low concentration of the predicted fatty acids in the samples might be the cause of this. The gas chromatograph could detect these compounds, but, their concentration is really near to the minimum detectable concentration of the system. Noise or small errors in the integration procedure can easily disrupt the results obtained. Finally, the lack of PLS factors make hard for these models to achieve a good prediction. It can be observed how these models show low RSQ value and, in some of them, high errors in cross-validation (SECV) and in external validation (SEP).

However, the second group of MPLS models with numerous PLS factors shown better results. These reference parameters are marked in bold in Table 3. Most of these models were developed for fatty acids with a higher concentration than the models described above. In these cases, RSQ>0.6 were obtained, sometimes reaching 0.8. SECV ranged from 5.3 to 23.0%. When external validation was carried out, quite good results were obtained for palmitic, margaric, oleic, linolelaidic, linoleic, and α-linolenic acids. Therefore, near infrared hyperspectral imaging also proved to be a promising tool for the screening of some individual fatty acids in walnut kernels.

Similar results to those obtained in the present study were obtained for total fat in Yi et al. (2017). However, these authors used a traditional NIR spectroscope for measuring powder walnut. No more studies have been found for walnuts. However, similar errors (between 1 and 17%) are described in other studies developed in olive oil, peanuts, and almonds for total fat and SFA, MUFA, PUFA, and some individual fatty acids (Milinovic et al., 2019; Teixeira and Sousa, 2019).

### Application of the Models

The application of the MPLS models for the prediction of total fat, SFA, MUFA, PUFA, and individual fatty acids in the non-selected sample set was carried out with three different objectives: (1) to verify the general trends obtained from the chemical analyses previously performed for the calibration and validation sets, (2) to check the cultivar trends previously observed in the chemical analysis, and (3) to check the feasibility of the sample selection procedure. Thus:

1. When comparing these results (Table 4) with those chemically obtained for calibration and validation sets (Table 1), it can be observed that values are similar. For example, average values of SFA, MUFA, and PUFA for predicted samples were 9.52, 14.23, and 76.22%, respectively. These values are near to those chemically obtained (9.40, 14.43, and 76.16%, respectively).
2. The similarity described in (1) is good evidence that the predictions of the models were adequate. Nevertheless, an ANOVA was carried out using these predicted data to thoroughly test the applicability of these MPLS models. ANOVA made possible to check which trends appear in the non-selected set and then check whether these trends are similar to those observed in the calibration and validation sets. Similar significant differences were found for SFA, MUFA, PUFA, C16:0, C18:1 ω9c, C18:2 ω6c, and C18:3 ω3. However, there were more significant differences between the different varieties in non-selected set than in calibration and validation sets. These can be due to a lack of accuracy in the MPLS predictions or to the larger number of samples in the non-selected set (102 samples) than in the calibration and validation sets (43+20 samples). With a larger number of samples, differences between varieties can be easily detected and identified as significant differences by the ANOVA.
3. The results presented in (1) and (2) confirm that the sample selection procedure carried out in this study is feasible due to similar trends were observed in the obtained values for selected and non-selected samples. Therefore, this procedure has been efficiently used to develop the models reducing the number of samples summited to the chemical analysis. Similar conclusion was achieved for other matrixes when a same or similar sample selection was carried out (Nogales-Bueno et al., 2015; Baca-Bocanegra et al., 2018a,b, 2019).

In summary, total fat and fatty acid profiles of five different walnut varieties (“Chandler”, “Franquette”, “Howard”, “Lara”, and “Tulare”) produced in Alentejo region have been obtained. Although no significant differences in total fat have been found between these varieties, the assessment of the identity of walnut oil produced in this region and with these varieties will be of major importance to protect the local walnut production. Significantly higher amounts of SFA and MUFA and a lower concentration of PUFA were found for “Franquette” than for the remaining varieties. These results may translate into greater oxidative stability and slightly lower health benefits of “Franquette” walnuts and oil comparing with other varieties. Therefore, “Franquette” variety is more suitable for oil production or for kernel sale whereas the remaining varieties are more suitable for in-shell sale.

In addition, MPLS models have been developed from the near infrared hyperspectral data and from the chemical data (total fat, SFA, MUFA, PUFA, and individual fatty acids) of walnuts. In general, good results were obtained in cross and external validations. Hyperspectral imaging is a promising tool for the screening of total fat, SFA, MUFA, PUFA, and some fatty acids. Using a set of samples additional to the validation set, the trends found in the fatty acid profiles of the different varieties, the applicability of the MPLS models, and the feasibility of the sample selection procedure carried out in this study have been verified.

Since gas chromatography is a relatively complex technique, requiring a large amount of different resources (time, chemical reagents, capital, etc.), the MPLS models obtained in this study acquire greater importance. In this study, first steps have been taken to develop robust, reliable, non-destructive, non-polluting, and rapid methods to control families of fatty acids and individual compounds in walnuts. More sets of samples from different walnut varieties, regions or seasons need to be collected and MPLS models need to be improved with these data. This would result in more reliable and robust methods. In addition, other studies using near-infrared hyperspectral imaging, such as control of tocopherols, triglycerides, etc. or detection of faulty samples, can be developed to help improve the walnut and walnut oil industry.

## Data Availability Statement

The original contributions presented in the study are included in the article/supplementary material, further inquiries can be directed to the corresponding author.

## Author Contributions

JN-B and AER: conceptualization. BB-B: methodology and visualization. JMB: software. JH-H and RG: validation. FH: formal analysis and funding acquisition. JN-B: investigation and writing – original draft preparation. AER: resources and supervision. JMB and RG: writing – review and editing. JH-H: project administration. All authors contributed to the article and approved the submitted version.

## Funding

This work was funded by National Funds through FCT – Foundation for Science and Technology under the Project UIDB/05183/2020 and by FEDER and National Funds through the Programa Operacional Regional ALENTEJO 2020 (ALT20-03-0246-FEDER-000064) – QualFastNut – Utilização da espectroscopia NIR para a análise rápida da qualidade em frutos secos.

## Conflict of Interest

The authors declare that the research was conducted in the absence of any commercial or financial relationships that could be construed as a potential conflict of interest.

## Publisher’s Note

All claims expressed in this article are solely those of the authors and do not necessarily represent those of their affiliated organizations, or those of the publisher, the editors and the reviewers. Any product that may be evaluated in this article, or claim that may be made by its manufacturer, is not guaranteed or endorsed by the publisher.
